# Pediatric-Onset Multiple Sclerosis in Tyrol, Austria: Epidemiology, Clinical Profile and Treatment Patterns from a Single Center (2015–2024)

**DOI:** 10.3390/children12121677

**Published:** 2025-12-10

**Authors:** Christian Lechner, Christopher Schoedl, Markus Breu, Alina Peternell, Matthias Baumann

**Affiliations:** 1 Pediatric Neurology, Department of Pediatric and Adolescent Medicine, Medical University of Innsbruck, 6020 Innsbruck, Austria; ch.lechner@i-med.ac.at (C.L.);; 2 Department of Neurology, Medical University of Vienna, 1090 Vienna, Austria

**Keywords:** pediatric-onset multiple sclerosis, pediatric neuro-immunological disorders, epidemiology, incidence, Austria

## Abstract

**Highlights:**

**What are the main findings?**

- This is the first population-based analysis of pediatric-onset multiple sclerosis (POMS) in Tyrol, Austria, covering a 10-year observation period (2015–2024).
- The pooled incidence was 2.19 per 100,000 person-years—among the highest reported in Europe—and the point prevalence at the end of the study period was 6.7 per 100,000 children.

**What are the implications of the main findings?**

- The high incidence situates Tyrol within the upper European range and supports an ongoing north–south gradient and increasing trend in POMS across high-income regions.
- Early recognition, comprehensive MRI/CSF diagnostics, and timely initiation of disease-modifying therapy enable favorable outcomes with minimal disability at transition to adulthood.

**Abstract:**

Background/Objectives: Pediatric-onset multiple sclerosis (POMS) accounts for 3–10% of all multiple sclerosis (MS) cases and represents the earliest manifestation of chronic autoimmune demyelination of the central nervous system. While increasing incidence rates have been reported in Europe, no regional data exist for Austria. This study aimed to determine the incidence, prevalence, and clinical characteristics of POMS in Tyrol, Austria, over a 10-year period. Methods: All patients diagnosed with MS before 18 years of age between 1 January 2015 and 31 December 2024, were included. Diagnoses were confirmed according to the 2010 and 2017 McDonald criteria and re-evaluated using the 2024 revisions. Demographic, clinical, cerebrospinal fluid (CSF), and MRI data were systematically reviewed. Incidence and prevalence rates were calculated with 95% confidence intervals (CI) using the exact Clopper–Pearson method. Results: Twenty-nine patients were newly diagnosed with POMS (26 female; median age 15 years, range 11–17), corresponding to a pooled 10-year incidence of 2.19 per 100,000 person-years (95% CI 1.47–3.14) and a point prevalence of 6.7 per 100,000 children (95% CI 3.1–12.8) as of 31 December 2024. Sensory (55%) and visual (31%) symptoms predominated at onset. Pleocytosis was found in 93%, and all CSF samples showed positive oligoclonal bands. MRI revealed supratentorial lesions in 97%, infratentorial in 69%, and spinal in 73%. The median annualized relapse rate was 0.67, and all patients had an EDSS of 0 at last follow-up. Conclusions: This first regional analysis of pediatric-onset MS in Austria demonstrates a higher incidence than most previously reported European cohorts, positioning Tyrol within the upper European range. The findings highlight the impact of early recognition, structured diagnostics, and timely initiation of disease-modifying therapy on favorable short-term outcomes.

## 1. Introduction

Pediatric-onset multiple sclerosis (POMS), defined by disease onset before 18 years of age, accounts for approximately 3–10% of all multiple sclerosis (MS) cases and represents the earliest manifestation of chronic autoimmune demyelination of the central nervous system [1,2,3,4]. Since children typically exhibit a more inflammatory disease course with higher relapse rates than adults, long-term disability tends to accumulate at a younger age, underscoring the need for early recognition and optimized management [5,6,7,8].

Over the past two decades, several population-based and registry studies have provided evidence for a rising incidence and prevalence of POMS worldwide [9,10,11,12,13,14]. Reported annual incidence rates range from 0.05 to 2.85 per 100,000 children, with regional variation across Europe, North America, and the Middle East [10,12,15,16,17,18,19,20]. Data from high-income regions, such as Canada, Northern Europe, and the United States, suggest increasing prevalence linked to improved diagnostics, changing environmental exposures, and potential latitude effects [9,11,21,22,23]. In Europe, recent national studies from Denmark, Poland, and Greece indicate incidence rates between 0.1 and 0.3 per 100,000 person-years [13,16,24].

In Austria, nationwide epidemiologic data on MS have shown a prevalence of approximately 98.5 per 100,000 inhabitants in 2002 and an increase to 158.9 per 100,000 in 2017, with an incidence of 19.5 per 100,000 person-years and a female-to-male ratio of about 2.5:1 [25,26]. However, data focusing specifically on pediatric-onset MS are lacking, and no regional analyses have been reported. Given the alpine geography, distinct healthcare structure, and well-defined population base, Tyrol provides an optimal setting for regional MS surveillance.

The present study aims to describe the epidemiology and clinical characteristics of pediatric-onset MS in Tyrol, Austria, over a 10-year period, based on a single-center cohort. We sought to determine the regional incidence and prevalence, delineate demographic and clinical features at onset, and provide context within the evolving European and global epidemiologic landscape of POMS.

## 2. Materials and Methods

### 2.1. Setting

Austria is a landlocked country in Central Europe with an area of 83,879 km^2^ and a total population of 9,159,993 as of 1 January 2024, by Statistik Austria. Compared with 8,584,926 on 1 January 2015, this corresponds to an absolute increase of 575,067 persons (+6.7%) over nine years. The federal state of Tyrol, located in the western part of the country, covers 12,648 km^2^, representing 15% of the Austrian territory.

Its resident population on 1 January 2024, was 775,970, of whom 133,854 (17.2%) were under 18 years of age, up from 728,826 on 1 January 2015, among them 130,179 minors (17.9%), i.e., an increase of 47,144 persons (+6.5%).

To our knowledge, official Austrian census data do not include information on the ethnic composition of the population; however, the majority of inhabitants are of Central European (Caucasian) origin. Health care in Austria is provided through a universal public health insurance system with unrestricted access to primary and specialist medical care. Neurological care for children and adolescents is regionally organized, with the Department of Pediatrics I at the Medical University of Innsbruck serving as the tertiary referral center for pediatric demyelinating diseases across the Tyrolean region.

### 2.2. Patient Recruitment

For this study, we included all patients diagnosed with MS between 1 January 2015, and 31 December 2024. All were part of our ongoing BIOMARKER study, a prospective multicenter cohort initiated in 2009 that currently comprises over 1300 children and adolescents referred for their first event of an acquired demyelinating syndrome (ADS).

### 2.3. Study Population and Diagnostic Criteria

Patients included in this study had to meet the following inclusion criteria: (1) diagnosis of MS fulfilling the 2024 revisions of the McDonald criteria [24], (2) age below 18 years at disease onset, and (3) written informed consent. Exclusion criteria included the diagnosis of another type of ADS like neuromyelitis optica spectrum disorders (NMOSD), myelin oligodendrocyte glycoprotein (MOG) antibody-associated disorders (MOGAD), or an infectious, metabolic, vascular, or neoplastic central nervous system (CNS) disease. Only patients with primary residence in Tyrol at the time of MS diagnosis were included in the epidemiologic analyses.

### 2.4. Demographic, Clinical and Laboratory Features

Demographic and anthropometric information, including age at onset, sex, and ethnicity, were extracted from the electronic medical records. Clinical manifestations at presentation—and, when applicable, those preceding the first evaluation—were categorized into five symptom domains: sensory (e.g., hypoesthesia), motor (e.g., weakness), visual (e.g., reduced visual acuity), cerebellar (e.g., ataxia), and other. Magnetic resonance imaging (MRI) findings, cerebrospinal fluid (CSF) analyses, and relevant blood studies were reviewed for all patients. Acute treatments as well as both initial and current disease-modifying therapies (DMTs) were documented [27,28].

### 2.5. Standard Protocol Approvals, Registrations and Patient Consents

The study was approved by the Ethics Committee of the Medical University of Innsbruck, Austria 151 (Study number AN4095). All patients and/or their caregivers provided written informed consent.

### 2.6. Statistical Methods

All 95% confidence intervals (95% CI) for prevalence and incidence rate estimates were calculated using the exact Clopper–Pearson method based on the binomial distribution. Quantitative variables were described using median and range. Statistical analysis was performed using IBM SPSS, release V.30.0 (IBM Corporation, Armonk, NY, USA).

## 3. Results

### 3.1. Epidemiology and Patient Demographics

During the 10-year observation period, a total of 29 patients were newly diagnosed with multiple sclerosis according to the 2010 and 2017 McDonald criteria and re-evaluated using the 2024 revision for this study. All patients had their primary residence in Tyrol at the time of diagnosis.

Annual incidence rates ranged from 0.8 to 5.3 per 100,000 children, with a median of 1.9 and a mean of 2.2 per 100,000. Using the exact Clopper–Pearson method, 95% confidence intervals for annual incidence rates ranged from 0.02–4.27 (2015, 1 case) to 2.13–10.90 (2019, 7 cases) per 100,000 children.

The pooled 10-year incidence across all observation years was 2.19 per 100,000 children (95% CI 1.47–3.14), based on 29 cases among approximately 1.3 million person-years at risk (see Table 1). As of 31 December 2024, nine children and adolescents under 18 years of age were living in Tyrol with a confirmed diagnosis of POMS, corresponding to a point prevalence of 6.7 per 100,000 (95% CI 3.1–12.8).

Ninety percent (26/29) of patients were female and 10% (3/29) male. The median age at diagnosis was 15 years (range 11–17). The median body mass index (BMI) was 22.1 kg/m^2^ (range 13.4–44.7). Based on age-adjusted growth charts, 6 patients (21%) were classified as obese and 4 (14%) as overweight. Twenty-eight patients (97%) were White, and one patient was of Near or Middle Eastern origin. Two patients (7%) reported a family history of multiple sclerosis; in both cases, the affected relative was the patient’s mother (see Table 2).

### 3.2. Clinical Characteristics at Baseline

The median time between onset of first symptoms and diagnosis was 10 days (range 1–218). In 16/29 patients (55.2%), disease onset presented with sensory symptoms, 9/29 (31.0%) had visual, 3/29 (10.3%) motor, and 1/29 (3.4%) cerebellar symptoms. The majority of the included patients, 17/29 (59%) experienced a monosymptomatic onset, the remaining 12/29 (41%) patients developed further symptoms during their first event.

At baseline, CSF analysis was performed in 27 of 29 patients (93%); two patients declined lumbar puncture despite medical advice. The median CSF cell count was 13/µL (range 3–69), and pleocytosis (≥4 cells/µL) was present in 25 of 27 patients (93%). Oligoclonal bands were detected in all analyzed samples (see Table 3 for further details).

To rule out other differential diagnoses, a broad laboratory work-up was done in these patients, including testing for antibodies against MOG and aquaporin-4. All 29 patients were negative for these two antibodies.

All patients underwent cranial MRI at baseline; in 26 of 29 (90%) cases, complete spinal imaging was additionally performed. MRI criteria for both dissemination in space (DIS) and dissemination in time (DIT) were fulfilled in 20 patients (69%), whereas 8 (28%) met only the DIS criterion, and 1 patient (3%) showed neither DIS nor DIT.

**Figure 1:**
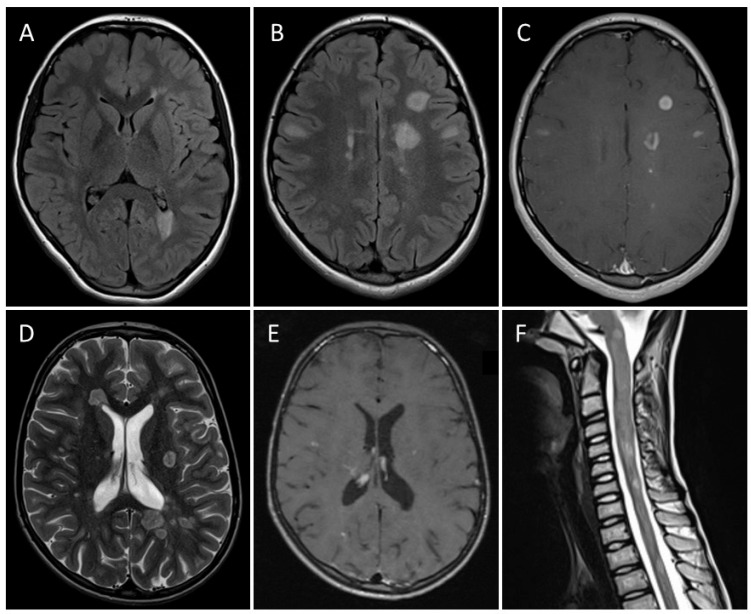
(**A**–**C**): Cerebral MRI of 14-year-old female showing multiple white matter lesions ((**A**,**B**), FLAIR sequence) with gadolinium enhancement ((**C**), T1w with Gd). (**D**–**F**): Cerebral and spinal MRI of 11-year-old male with multiple supratentorial white matter lesions ((**D**), T2w) of whom some show gadolinium enhancement ((**E**), T1w with Gd). Several spinal lesions are also visible ((**F**), T2w).

Supratentorial lesions were detected in 28/29 (97%), infratentorial in 20/29 (69%), and spinal in 19/26 (73%) (see Table 4 for further details; see Figure 1 for representative MRI).

### 3.3. Follow-Up

Median follow-up time from diagnosis to either the 18th birthday or the end of observation period (31 December 2024) was 2.0 years (range 0.1–5 years). In total, 61 patient-years were analyzed, during which 54 relapses occurred. The median number of relapses per patient until age 18 or study end was 2, corresponding to a median annualized relapse rate (ARR) of 0.67 (range 0.20–3.0). The Expanded Disability Status Scale (EDSS) score was 0 for 28/29 patients and 1 for the remaining patient at the last follow-up (either at 18 years of age or at the end of the study). All patients showed a relapsing-remitting disease course throughout the observation period.

### 3.4. Treatment Details

Each patient’s first clinical event was treated with high-dose intravenous methylprednisolone according to standard protocol. Twenty-seven patients (93%) commenced DMT (interferon-β 16/27; dimethyl fumarate 6/27; fingolimod 5/27), while two patients experienced only one clinical event during follow-up and did not start long-term treatment contrary to medical recommendation. At the end of follow-up or upon reaching 18 years of age, 8/ 27 patients (30%) remained on their initial DMT (interferon-β 3/8; dimethyl fumarate 3/8; fingolimod 2/8). Two patients discontinued therapy due to adverse effects despite medical advice.

The remaining seventeen patients (63%) had to switch therapy at least once either due to relapse (*n* = 10), radiological progress without clinical symptoms (*n* = 10) or side effects (*n* = 5). Subsequent treatments included dimethyl fumarate (9/17), teriflunomide (4/17), natalizumab (2/17), ocrelizumab (1/17) or fingolimod (1/17). Among these, 7/17 (41%) required an additional switch, most frequently to fingolimod (5/7), ofatumumab (1/7), or natalizumab (1/7). One patient experienced another relapse under her third medication —after interferon-β, teriflunomide, and fingolimod— and was subsequently switched to natalizumab.

## 4. Discussion

This 10-year, population-based analysis represents the first epidemiological and clinical characterization of POMS in Tyrol, Austria. Between 2015 and 2024, a total of 29 children and adolescents were newly diagnosed with MS, resulting in a pooled incidence of 2.19 per 100,000 (95% CI 1.47–3.14) and a point prevalence of 6.7 per 100,000 inhabitants below the age of 18 years at the end of the observation period. The median age at diagnosis was 15 years, with a marked female predominance (female-to-male ratio 8.7:1). These figures exceed most previously reported European POMS incidence rates (0.45–0.79/100,000) and, by that, reinforce previous observations of a rising trend across high-income regions [6,7,12,15].

Comparable studies from Denmark [6], Poland [9], Greece [10], and Slovenia [11] have documented incidences between 0.66 and 0.79 per 100,000. The highest European pediatric incidence has been reported from Sardinia [19] (2.85 per 100,000; comparable age band and McDonald-based case definition). Our rates approach this upper bound, placing Tyrol in the upper European range. This is consistent with the general increase in Austrian MS prevalence from 98.5/100,000 in 2002 [25] to 158.9/100,000 in 2017 [26]. A major limitation when comparing our data with those from neighboring Germany is that Reinhardt et al. included only patients aged ≤15 years.

Together, these data indicate that environmental and diagnostic factors—rather than genetic predisposition alone—likely contribute to observed regional differences.

The markedly female-predominant sex distribution in our cohort warrants consideration of several factors. First, the small underlying population denominator in Tyrol (~130,000 children under 18 years) inherently leads to statistical instability; with a total of only 29 cases across 10 years, even minimal absolute differences (e.g., 3 boys vs. the 6 expected based on European data) can substantially shift the female-to-male ratio. Second, as our center is the sole tertiary referral institution for pediatric demyelinating disorders in Tyrol, all newly presenting cases should theoretically be captured within our system, minimizing the likelihood of under-ascertainment. If referral bias were present, it would more plausibly favour the inclusion of male patients, as boys are known to present with more severe initial attacks and are therefore more likely to be referred early making a referral-driven female overrepresentation unlikely. Third, non-MS demyelinating disorders, e.g., MOGAD, which might show more balanced or even male-leaning sex distributions, were strictly excluded may further accentuate the female predominance within a small MS-only cohort. Taken together, these factors suggest that the observed sex ratio is more likely a consequence of population size and diagnostic stratification rather than a true epidemiologic deviation. While this limits the generalizability of the sex distribution, it does not affect the epidemiologic incidence and prevalence estimates, which are based on the entire pediatric population at risk.

Clinically, the predominance of sensory (55%) and visual (31%) onsets and the low proportion of cerebellar or motor involvement are consistent with most European pediatric MS cohorts [1,2,7]. The short latency between symptom onset and diagnosis (median 10 days) underscores the effectiveness of regional diagnostic structures and increased awareness. CSF findings closely mirror previous data: pleocytosis was present in 93%, and all tested samples showed positive oligoclonal bands, confirming intrathecal B-cell activation as a near-universal feature of early MS [29]. Of note, two patients with available kappa free light chain (KFLC) indices showed strongly elevated values, supporting recent evidence that KFLC may serve as a sensitive diagnostic marker complementing or even replacing the IgG index [24,29].

MRI features fulfilled both dissemination in space and time in 69% of cases, comparable to reports from Denmark [6] and the Netherlands [8]. Supratentorial lesions were almost universal (97%), while infratentorial and spinal lesions occurred in 69% and 73%, respectively—figures that parallel other Northern and Central European cohorts [7,9,11]. The inclusion of both cranial and complete spinal imaging in nearly all patients further substantiates the diagnostic robustness of this single-center study.

The median annualized relapse rate (ARR) of 0.67 over 61 patient-years and the absence of permanent disability (EDSS 0 in 28/29 patients, 1 in 1/29 at last follow-up) are encouraging findings. Despite the occurrence of clinical relapses and radiological activity (without clinical symptoms), almost all patients had an EDSS score of 0 at last follow-up. This finding is consistent with previous POMS literature demonstrating that children often recover completely after relapses and accumulate disability much more slowly than adults [30,31]. The EDSS further underestimates disability in pediatric patients because it is relatively insensitive to visual, cognitive, and subtle motor deficits. Therefore, low EDSS scores do not contradict the presence of relapse activity or the need for treatment escalation. Consequently, although pediatric MS is typically more inflammatory than adult-onset disease [2,4], early diagnosis and structured DMT initiation appear to mitigate long-term functional decline. The predominance of relapsing–remitting courses in all patients matches other pediatric MS cohorts, where progressive disease before adulthood remains exceptional [1,31].

Therapeutically, our data reflects the evolving European treatment paradigm toward early high-efficacy therapy [3,32]. Interferon-β remained the most frequent initial DMT (59%)—at least prior to the EMA approval of newer agents such as fingolimod (2018) and dimethyl fumarate (2022)—followed by dimethyl fumarate (22%) and fingolimod (19%). However, 63% of patients required at least one switch, most often due to relapse or radiologic progression. Fingolimod, natalizumab, and anti-CD20 therapies (ocrelizumab, ofatumumab) emerged as predominant high- or very high-efficacy treatment options, consistent with current practice trends in Western Europe and Canada [23,33]. The high switching rate may reflect both the more aggressive inflammatory phenotype in pediatric patients and increasing clinician confidence in escalation or early intensive approaches [34].

Several factors must be considered when interpreting this data. The monocentric setting ensures diagnostic consistency but limits generalizability. Given Tyrol’s population size, annual incidence estimates are subject to natural statistical fluctuation; nevertheless, the 10-year pooled rate provides a stable indicator of regional disease burden. The prospective inclusion within a long-standing biomarker study adds further validity. Finally, while our follow-up duration was relatively short, the uniform absence of disability at transition to adulthood highlights the favorable outcomes achievable under early and structured care.

## 5. Conclusions

In conclusion, this study establishes baseline epidemiologic and clinical data for pediatric-onset MS in Tyrol, Austria, positioning the region within the upper European incidence range. The findings reinforce the critical role of early recognition, comprehensive MRI and CSF evaluation, and timely DMT initiation. Ongoing surveillance and integration into multinational registries will be essential to validate these trends and to further refine early treatment strategies in pediatric MS.

## Figures and Tables

**Table 1:** Incidence of POMS between 2015 and 2024.

Year	Patients	Pediatric Population	Incidence/100,000	95% CI (Exact, Clopper–Pearson)
2015	1	130,179	0.77	0.02–4.27
2016	2	131,047	1.53	0.19–5.50
2017	2	132,001	1.52	0.18–5.49
2018	2	132,259	1.51	0.18–5.45
2019	7	132,432	5.29	2.13–10.90
2020	4	132,412	3.02	0.82–7.73
2021	3	133,091	2.25	0.46–6.56
2022	2	134,174	1.49	0.18–5.39
2023	4	133,854	2.99	0.81–7.66
2024	2	133,854	1.49	0.18–5.39

**Table 2:** Patient demographics.

Parameter	Results
Median age in years (range)	16 (11–17)
Median height in cm (range)	163 (147–182)
Median weight in kg (range)	62.2 (29–110.5)
Median BMI in kg/m^2^ (range)	22.1 (13.4–44.7)
Female	26/29
Race and ethnicity	28/29 White, 1/29 Near or Middle East
Family history of MS	2/29

**Table 3:** Cerebrospinal fluid analyses.

Parameter	Results (Range)
Median cell count (cells/µL)	13 (3–69)
Pleocytosis (≥4 cells/µL)	25/27
Positive oligoclonal bands	27/27
Median total CSF protein (mg/dl)	35.4 (20.4–115)
Median glucose ratio	0.63 (0.52–0.85)
Median IgG index ^1^	0.99 (0.51–3.24)
Kappa free light chain index ^2^	30.77 and 277.49

^1^ Available in 25/27 patients. ^2^ Available in 2/27 patients.

**Table 4:** MRI details.

Parameter	Results
Supratentorial lesions	28/29
Infratentorial lesions	20/29
Spinal lesions	19/26
Supra-, infratentorial and spinal lesions	14/26
Periventricular	24/29
Corpus callosum	10/29
Cortical (including sub- and juxtacortical)	22/29
Deep White Matter	13/29
Deep Grey Matter	2/29
Optic nerve	4/29
Brainstem	14/29
Cerebellum	15/29
Spinal cord—cervical	17/26
Spinal cord—thoracic	6/26

## Data Availability

The data presented in this study is available on request from the corresponding author. The data are not publicly available due to legal reasons.

## Abbreviations

The following abbreviations are used in this manuscript:

ADS	Acquired demyelinating syndrome
ARR	Annualized relapse rate
BMI	Body mass index
CI	Confidence interval
CNS	Central nervous system
CSF	Cerebrospinal fluid
DIS	Dissemination in space
DIT	Dissemination in time
DMT	Disease-modifying therapy
EDSS	Expanded Disability Status Scale
EMA	European Medicines Agency
Gd	Gadolinium
IgG	Immunoglobulin G
KFLC	Kappa free light chain
MOG	Myelin oligodendrocyte glycoprotein
MOGAD	Myelin oligodendrocyte glycoprotein antibody-associated disorders
MRI	Magnetic resonance imaging
MS	Multiple sclerosis
NMOSD	Neuromyelitis optica spectrum disorders
POMS	Pediatric-onset multiple sclerosis
SPSS	Statistical Package for the Social Sciences
